# Patterns of Referral for Fertility Preservation Among Female Adolescents and Young Adults with Breast Cancer: A Population-Based Study

**DOI:** 10.1089/jayao.2018.0102

**Published:** 2019-04-05

**Authors:** Ann Korkidakis, Katherine Lajkosz, Michael Green, Donna Strobino, Maria P. Velez

**Affiliations:** ^1^ Division of Reproductive Endocrinology and Infertility, Department of Obstetrics and Gynecology, Queen's University, Kingston, Canada.; ^2^ Division of Reproductive Endocrinology and Infertility, University of British Columbia, Vancouver, Canada.; ^3^ Institute for Clinical Evaluative Sciences, Queen's University, Kingston, Canada.; ^4^ Department of Family Medicine, Centre for Health Services and Policy Research, Queen's University, Institute for Clinical Evaluative Sciences, Kingston, Canada.; ^5^ Department of Population, Family, and Reproductive Health, Bloomberg School of Public Health, Baltimore, Maryland.

**Keywords:** oncofertility, fertility preservation, breast cancer, premature ovarian insufficiency

## Abstract

***Purpose:*** To assess the fertility preservation (FP) referral rates and patterns of newly diagnosed breast cancer in female adolescent and young adult (AYA) patients.

***Methods:*** Women aged 15–39 years with newly diagnosed breast cancer in Ontario from 2000 to 2017 were identified using the Ontario Cancer Registry. Exclusion criteria included prior sterilizing procedure, health insurance ineligibility, and prior infertility or cancer diagnosis. Women with a gynecology consult between cancer diagnosis and chemotherapy commencement with the billed infertility diagnostic code (ICD-9 628) were used as a surrogate for FP referral. The effect of age, parity, year of cancer diagnosis, staging, income, region, neighborhood marginalization, and rurality on referral status was investigated.

***Results:*** A total of 4452 patients aged 15–39 with newly diagnosed breast cancer met the inclusion criteria. Of these women, 178 (4.0%) were referred to a gynecologist with a billing code of infertility between cancer diagnosis and initiation of chemotherapy. Older patients, prior parity, and advanced disease were inversely correlated with referrals. Referral rates also varied regionally: patients treated in the south-east and south-west Local Health Integration Networks (LHINs) had the highest probability of referral, and patients covered by north LHINs had the lowest (central LHIN as reference). General surgeons accounted for 36.5% of all referrals, the highest percentage of all specialists. Referral rates significantly increased over time from 0.4% in 2000 to 10.7% in 2016.

***Conclusion:*** FP referral rates remain low and continue to be influenced by patient demographics and prognosis. These findings highlight the need for further interdisciplinary coordination in addressing the fertility concerns of AYA with newly diagnosed breast cancers.

## Background

Breast cancer is the most commonly diagnosed malignancy among Canadian women of reproductive age.^1^ Given advancements in therapeutic options, survival rates continue to improve,^2^ and achieving a cure is no longer the sole goal. Efforts are now directed toward addressing the physical and psychological impact of both the disease process as well as the associated treatment.^3^ Fertility potential has a major impact on the quality of life of adolescent and young adult (AYA) cancer survivors.^4^ With the emergence of oncofertility, an interdisciplinary field aimed at addressing the reproductive needs of oncology patients, innovative technologies have been developed to provide fertility preservation (FP) treatments in patients undergoing gonadotoxic therapy. Nonetheless, patient referral rates continue to be staggeringly low.^5–9^

Fertility may be significantly compromised in women following breast cancer treatment. Chemotherapeutic agents often have gonadotoxic effects.^10,11^ While infertility may be a secondary concern during the period of active treatment, it has a significant impact on the quality of life of cancer survivors. In a recent survey, female cancer patients aged 15–45 years rated the impact of cancer treatment on fertility to be of utmost importance.^12^ Those who experience infertility as a result of treatment have depression, grief, and sexual dysfunction.^3,13^

Emphasis has been placed on shortening FP protocols to permit prompt initiation of cancer treatment. Delays in referrals, however, can significantly compromise the success and feasibility of these treatments.^14^ Results of a prospective study of breast cancer patients demonstrated that expedited referrals allow for earlier commencement of cryopreservation as well as subsequent chemotherapy. Additionally, women referred earlier were more likely to complete multiple FP cycles and ultimately had a greater number of oocytes or embryos that were cryopreserved.^15^

The American Society of Clinical Oncology recently updated guidelines recommending that a discussion about FP should be initiated with all reproductive-aged patients at risk of infertility as a consequence of treatment.^16^ The society endorsed oncofertility referrals for all interested patients and specified that even ambivalent patients should be referred for additional discussion. Despite these recommendations, numerous studies report very low rates of discussion between patients and health care providers regarding the gonadotoxic effects of cancer treatment on fertility.^5–9^ Furthermore, a significant proportion of the dialogue is prompted by patients rather than health care providers.^17^ FP referrals should be encouraged as breast cancer patients seemingly prefer to receive fertility-related information from reproductive endocrinologists.^18^

The current literature on determinants of FP referrals is mostly limited to small qualitative studies.^18–23^ Available Canadian data about referrals is derived from analyses of surveys.^22^ A crucial step in improving patient access to FP services is identification of the barriers impeding timely referral to oncofertility. Accordingly, the purpose of this study was to investigate the provincial referral patterns of AYAs with newly diagnosed breast cancer for a fertility assessment through a population-level approach.

## Materials and Methods

### Study design and population

Our retrospective, population-based cohort study describes fertility referral rates and patterns among female AYAs with newly diagnosed breast cancer who were receiving chemotherapy in Ontario, Canada. Ontario is the country's largest province, with a population of ∼13.2 million. Women aged 15–39 years with an initial diagnosis of breast cancer between January 2000 and September 2017 who received chemotherapy were eligible for the study. This age range was selected because the U.S. National Cancer Institute defines the age of AYAs with cancer to be between 15 and 39 years.^23^ Exclusion criteria were a history of a sterilizing procedure, ineligibility for provincial health insurance coverage at the time of diagnosis, diagnosis of infertility at any point before breast cancer diagnosis, any other prior cancer diagnosis, or a breast cancer diagnosis before 2000. Sterilizing procedures included prior hysterectomy, bilateral oophorectomy, or tubal ligation.

### Data sources

Data for the study were acquired from the Institute for Clinical Evaluative Sciences (ICES)'s electronic health care administrative databases. The incident cohort was established through the Ontario Cancer Registry (OCR). The registry is a comprehensive provincial database that captures at least 98% of incident cancers in Ontario.^24,25^ In addition to defining the cohort, the OCR provided diagnostic information.

The OCR data were linked to several health care data sources. Information on referrals was obtained from the Ontario Health Insurance Plan (OHIP) database, which captures all paid billing claims for physician ambulatory visits across the province. Demographic and eligibility information was obtained from the Registered Persons Database (RPDB), a roster of all OHIP beneficiaries. Parity at cancer diagnosis was identified using the MOMBABY dataset, which contains inpatient admission records of mothers with a live birth between fiscal years 1988 and 2017. Physician characteristics were obtained using the ICES Physician Database, which contains yearly information about all physicians in Ontario. The datasets were linked using unique encoded identifiers. Analyses of the data were conducted at the ICES.

### Exposures and outcomes

The primary outcome, referral for a discussion on FP between the diagnosis of cancer and commencement of chemotherapy treatment, was identified through a gynecology consult (OHIP billing code A205) with a billed diagnostic code of infertility (ICD-9 628) occurring during that specific time interval. Women with a referral to gynecology without a diagnosis of infertility were excluded from the analysis. Women were followed until death, loss of OHIP eligibility, or February 28, 2018, whichever occurred first. Diagnostic OCR data were available for only part of 2017; it was excluded from analysis of temporal trends.

### Patient characteristics

Age at cancer diagnosis was categorized as 15–29 and 30–39 years based on the differing age limits for AYA with cancer. The Canadian Cancer Society sets the upper limit at 29 years, while the U.S. National Cancer Institute includes individuals up to 39 years.^23,26,27^ Women with a prior live birth at the time of cancer diagnosis were also identified. Time from breast cancer diagnosis to start of chemotherapy was subdivided at ≤6 weeks and >6 weeks based on Cancer Care Ontario's target of time from referral to systemic chemotherapy of ≤6 weeks (referral to visit target = 14 days; visit to systemic treatment target = 28 days).^28^ Over 75% of cancer centers in Ontario met this target in 2017.

Several covariables were studied. The Ontario neighborhood marginalization score is a census-based, area-level measure of socioeconomic status that has been shown to be associated with health outcomes and behaviors.^29,30^ In the province, there are 13 regional cancer centers that were grouped into four regions based on their corresponding provincial Local Health Integration Networks (LHINs).^31^ Neighborhood marginalization was categorized as a dichotomous variable defined by the 0–60th and 61–100th percentiles, with a higher percentile denoting greater marginalization. Income quintile measured household size-adjusted income based on census data and used postal codes to rank the average neighborhood among other neighborhoods in the census area. Income quintile was categorized as 0–60th and 61–100th percentile, with a higher percentile denoting higher average income. Residence was defined as either rural (community size <10,000) or urban.

### Statistical analysis

Descriptive statistics were used to compare referral status by patient characteristics. Differences in referral rates by patient characteristics were assessed using chi-square analysis for categorical variables, and Student *t*-test for continuous variables. Annual trends in the proportion of breast cancer patients with a referral were assessed using the Cochran-Armitage test.

Univariable logistic regression models were used to examine associations between patient characteristics and referral status. A multivariable analysis was conducted to adjust for those variables that were significant in the univariable analysis: age, parity, year of diagnosis, and region. Disease stage was not included since it was missing in a large portion of the sample. Risk ratios with 95% confidence intervals and *p*-values were calculated. Data were analyzed using SAS version 9.3 (Cary, North Carolina). All statistical tests were two-sided, and *p*-values <0.05 were considered statistically significant.

This study was approved by the Research Ethics Board at the Queen's University in Kingston, Ontario.

## Results

### Study population

A total of 4452 women aged 15–39 years who had an initial diagnosis of breast cancer between 2000 and 2016 met the inclusion criteria. Of these women, 178 (4.0%) received a gynecology consult with a billing code of infertility between their diagnosis and initiation of chemotherapy. The characteristics of the study population are reported in Table 1.

**Table 1.** Characteristics of Adolescents and Young Adults Diagnosed with Breast Cancer Who Were Treated with Chemotherapy Between 2000 and 2017 in Ontario

		*Gynecology referral before chemotherapy* n *(%)*	
*Characteristics*	*All cases (*N* = 4452)* n *(%)*	*Yes (*N* = 178)*	*No (*N* = 4274)*
Age at diagnosis^*^			
15–29	504 (11.3)	67 (37.6)	437 (10.2)
30–39	3948 (88.7)	111 (62.4)	3837 (89.8)
Marginalization quintile^a^			
0–60	2685 (60.3)	111 (62.4)	2574 (60.2)
61–100	1721 (38.7)	67 (37.6)	1654 (38.7)
Neighborhood income quintile^b^			
0–60	2535 (56.9.0)	95 (53.4)	2440 (57.1)
61–100	1901 (42.7)	83 (46.6)	1818 (42.5)
Rurality			
Urban	4037 (90.7)	163 (91.6)	3669 (90.6)
Rural	415 (9.3)	15 (8.4)	377 (9.4)
Prior parity^*^			
No	1847 (41.5)	142 (79.8)	1705 (39.9)
Yes	2605 (58.5)	36 (20.2)	2569 (60.1)
Cancer staging^c,^^*^			
I	160 (3.6)	7 (3.9)	153 (3.6)
II	396 (8.9)	<6	390 (9.1)
III	239 (5.4)	<6	237 (5.5)
IV	34 (0.8)	0	34 (0.8)
Time from diagnosis to chemotherapy			
≤6 weeks	1229 (27.6)	49 (27.5)	1180 (27.6)
>6 weeks	3223 (72.4)	129 (72.5)	3094 (72.4)
Region of oncology care^d,e,^^*^			
South central	1741 (39.1)	44 (24.7)	1697 (38.1)
North	296 (6.7)	<6	286–296
South-east	894 (20.1)	57 (32.0)	837 (19.6)
South-west	987 (22.2)	56 (31.5)	931 (21.8)

^a^ Marginalization quintile 0–60 represents the lowest 60% of marginalization. Marginalization data missing for 52 patients.

^b^ Neighborhood income quintile 0–60 represents the communities where the poorest 60% of the Ontario population reside. Neighborhood income quintile missing for 16 patients.

^c^ Cancer staging data only available for 18.6% of sample.

^d^ Region of oncology care consists of grouped LHINs. Regional data missing for 534 patients.

^e^ Variables with <6 patients have numbers suppressed as per ICES privacy policy.

^*^ *p* < 0.01.

ICES, Institute for Clinical Evaluative Sciences; LHINs, Local Health Integration Networks.

Women referred to gynecology for a fertility assessment were younger, had an early cancer stage, and were more commonly nulliparous as compared with women who were not referred. While the percentage that were referred was higher among women who lived in urban and higher-income neighborhoods, this finding was not statistically significant (*p* = 0.21). The proportion of women considered more marginalized was also not different between the referred and non-referred group.

### Referral rates and patterns

**FIG. 1.**
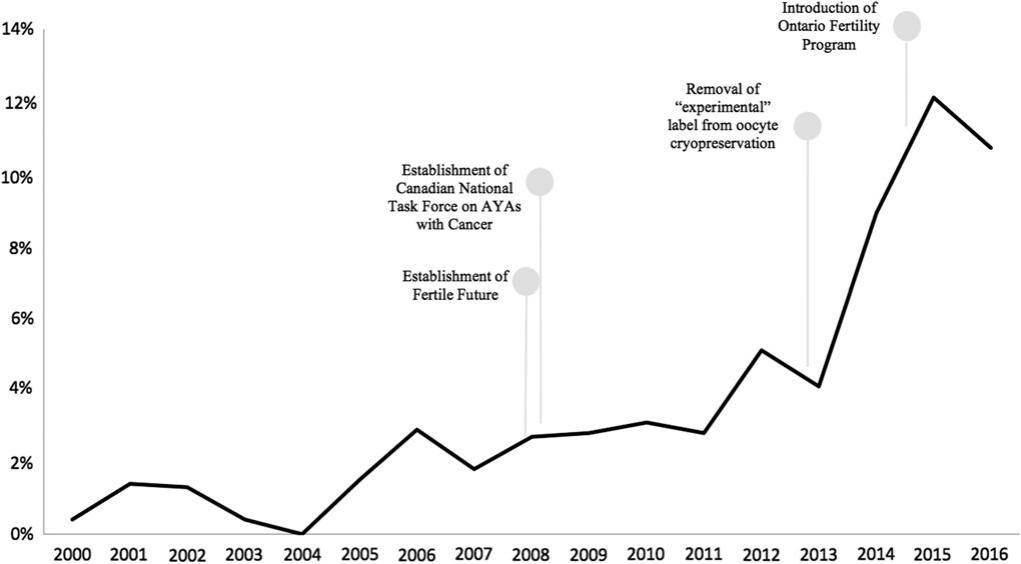
Percentage of eligible adolescents and young adults diagnosed with breast cancer who were referred for fertility assessment before chemotherapy in Ontario.

Overall, there was a trend toward an increase in FP referrals over time in Ontario, Canada (Fig. 1). In 2000, only 0.4% of newly diagnosed breast cancer patients meeting the study criteria were referred for a discussion on FP. This percentage rose to 10.7% in 2016 (*p* < 0.01).

**FIG. 2.**
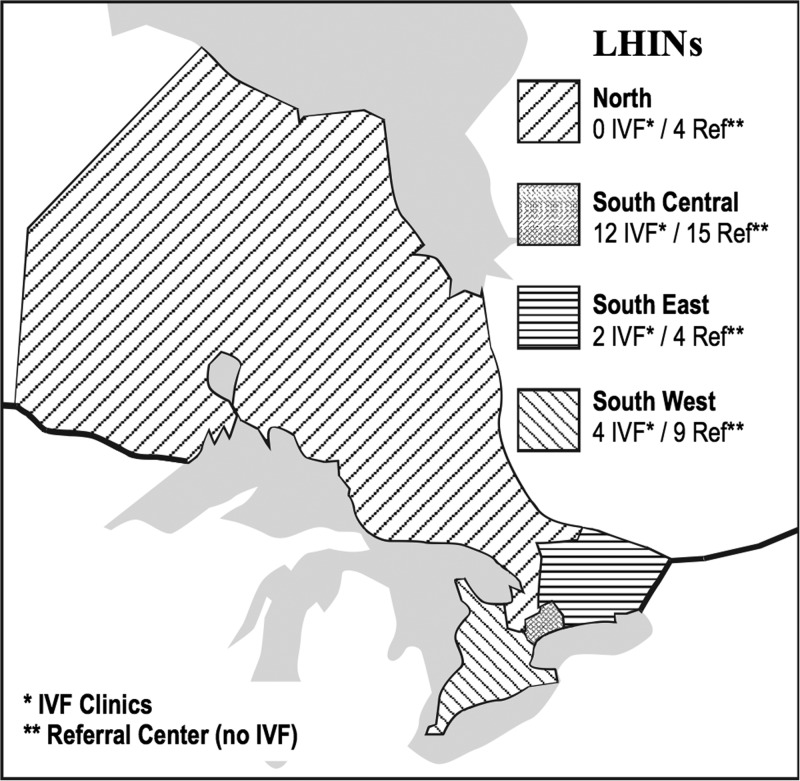
Distribution of fertility clinics among grouped LHINs. LHIN, Local Health Integration Network.

The results of logistic regression univariable analysis are shown in Table 2. Older age was associated with a reduced odds of referral. Prior birth was also negatively correlated with assessments. Women with a more advanced disease had lower chance of referral compared with women with stage I diagnosis. Referral rates also varied regionally, with patients treated in the south-east and south-west LHINs having the highest probability of referral and those managed in the north LHINs having the lowest compared with those managed in the central region. These regions are defined, along with the provincial distribution of *in vitro* fertilization (IVF) clinics and referral centers, in Figure 2. The odds of referral started to rise in 2006, but this trend accelerated in 2013. Age, parity, year of diagnosis, and some regions remained statistically significant after multivariable analysis (Table 3). Income quintile, marginalization, rurality, and time from cancer diagnosis to chemotherapy were not significantly associated with referral status in the univariable or multivariable analysis.

**Table 2.** Univariable Analysis

*Characteristic*	*Proportion assessed by fertility specialist %*	*OR (95% confidence interval)*	p
Age at diagnosis			
15–29 (ref)	13.3	1.0	
30–39	2.8	0.19 (0.14–0.26)	<0.01
Marginalization quintile^a^			
0–60 (ref)	4.1	1.0	
61–100	3.9	0.94 (0.69–1.28)	0.69
Neighborhood income quintile^b^			
0–60 (ref)	3.7	1.0	
61–100	4.4	1.17 (0.87–1.58)	0.30
Rurality			
Urban (ref)	4.0	1.0	
Rural	3.6	0.89 (0.52–1.53)	0.68
Prior live birth			
No (ref)	7.7	1.0	
Yes	1.4	0.17 (0.12–0.24)	<0.01
Cancer staging^c^			
I (ref)	4.4	1.0	
II	1.5	0.34 (0.11–1.02)	0.05
III	0.8	0.18 (0.04–0.90)	0.04
IV	—	—	
Year of diagnosis			
2000–2003 (ref)	0.9	1.0	
2004–2007	1.5	1.68 (0.73–3.86)	0.22
2008–2011	2.9	3.24 (1.52–6.87)	<0.01
2012–2016	8.3	9.96 (5.02–19.78)	<0.01
Time from diagnosis to chemotherapy			
≤6 weeks (ref)	4.0	1.0	0.98
>6 weeks	4.0	1.0 (0.72–1.40)	
Region of oncology care^d,e^			
South central (ref)	2.5	1.0	
North	<1	0.13 (0.02–0.95)	0.04
South-east	6.4	2.63 (1.76–3.93)	<0.01
South-west	5.7	2.32 (0.88–2.57)	<0.01

Determinants of fertility assessment before chemotherapy in AYAs diagnosed with breast cancer between 2000 and 2017 in Ontario.

^a^ Marginalization quintile 0–60 represents the lowest 60% of marginalization. Marginalization data missing for 52 patients.

^b^ Neighborhood income quintile 0–60 represents the communities where the poorest 60% of the Ontario population reside. Neighborhood income quintile missing for 16 patients.

^c^ Cancer staging data only available for 18.6% of sample.

^d^ Region of oncology care consists of grouped LHINs. Regional data missing for 534 patients.

^e^ Variables with <6 patients have numbers suppressed as per ICES privacy policy.

AYA, adolescent and young adult; OR, odds ratio.

**Table 3.** Multivariable Analysis

*Characteristic*	*OR (95% confidence interval)*	p
Age at diagnosis		
15–29 (ref)	1.0	
30–39	0.30 (0.21–0.43)	<0.01
Prior live birth		
No (ref)	1.0	
Yes	0.22 (0.15–0.32)	<0.01
Year of diagnosis		
2000–2003 (ref)	1.0	
2004–2007	1.56 (0.67–3.60)	0.30
2008–2011	3.15 (1.46–6.78)	<0.01
2012–2016	9.48 (4.71–19.06)	<0.01
Region of oncology care^a^		
South central (ref)	1.0	
North	0.17 (0.02–1.22)	0.08
South-east	3.44 (2.25–5.26)	<0.01
South-west	3.09 (2.02–4.73)	<0.01

Determinants of fertility assessment before chemotherapy in AYAs diagnosed with breast cancer between 2000 and 2017 in Ontario.

^a^ Region of oncology care consists of grouped LHINs. Regional data missing for 534 patients.

### Referring physician

The characteristics of referring physicians were analyzed (Table 4). General surgeons were responsible for the largest percentage of FP referrals for breast cancer patients (36.5%), followed by medical oncologists (27.0%) and family physicians (20.8%). The difference in referral rates among medical specialties was statistically significant (*p* < 0.01). No differences were observed according to physician sex and age.

**Table 4.** Characteristics of Physicians Referring Eligible Adolescents and Young Adults with Breast Cancer for Fertility Assessment Before Chemotherapy Between 2000 and 2017 in Ontario

*Characteristics*	N* = 178,* n *(%)*	p
Age		0.40
≤45	94 (52.8)	
>45	80 (44.9)	
Sex		0.07
Female	105 (59.0)	
Male	71 (39.9)	
Specialty		<0.01
Family medicine	37 (20.8)	
General surgery	65 (36.5)	
Medical oncology	48 (27.0)	
Other	16 (11.3)	

## Discussion

The results of this large, population-based retrospective study of female AYA patients with breast cancer indicate several important referral trends and patterns. While the referral rates for fertility assessments in our study were remarkably low, they are consistent with prior findings. The results of a retrospective cohort study of 793 eligible breast cancer patients treated at Northwestern Memorial Hospital showed that only 4% of patients were referred to oncofertility at baseline.^32^ A similar study conducted at the Massachusetts General Hospital reported referral rates between 1.7% and 3% for reproductive-aged women with any cancer diagnosis.^33^ Data from Europe demonstrated slightly higher referrals rates, up to 9.8% of all female cancer patients.^34^ Prior Canadian data are limited to survey analysis citing very low numbers of monthly clinic referrals.^1^

There have been notable changes to the field of oncofertility that overlap with the increase in FP referrals starting in 2008, with particularly steep rise from 2013 to 2015. Two important national initiatives were established in 2008. The Canadian National Task Force on Adolescents and Young Adults with Cancer was formed that year. Notably, oncofertility was identified as a priority issue in improving the quality of life of AYA cancer survivors.^35,36^ Additionally, Fertile Future, a national non-profit organization, was established. To date, this organization has financially contributed toward the FP of over 500 Canadians, with numerous others benefiting from their educational resources. In early 2013, the “experimental” label was officially removed from oocyte cryopreservation.^37^ This change was prompted by technological advances in the freezing of human eggs that led to significant improvement in subsequent pregnancy rates. Since that time, women are no longer required to have a male partner or use donor sperm to participate in FP. These national and international-level changes have facilitated access to a range of FP options.

Neighborhood income quintile and marginalization were not found to be a significant determinant of FP referral. This finding may be specific to the public health system in Canada. Currently, medical consultation with oncofertility specialists is covered by public health insurance in all Canadian provinces. Financial barriers may be more evident when examining the rates of FP procedures. The costs of FP are likely prohibitive to a significant proportion of Canadian women, as they can surpass 10,000$ CAD. Importantly, up to the end of 2015, only women with bilateral tubal blockage received funding for assisted reproductive treatments in Ontario. The Ontario Fertility Program, introduced in December 2015, covers the cost of one IVF cycle per lifetime for all female patients under the age of 43, regardless of family status or reason for fertility treatment. Cancer patients seeking FP are eligible for coverage if they hold a valid OHIP card. Notably, our findings show a slight decline in referral rates from 2015 to 2016, highlighting the importance of further exploring the effect of provincial IVF coverage on FP referrals and procedures.

In this study, patient interest in FP could not be assessed and may potentially account for the low referral rates. Stress related to cancer diagnosis or upcoming treatment may deter patients from pursuing elective FP procedures.^12,38^ Importantly, the literature suggests that up to 75% of reproductive-aged women diagnosed with cancer are interested in future childbearing.^38^ Furthermore, formal recommendations urge physicians to refer even ambivalent patients for an oncofertility consult.^16^ As fertility potential has a large impact on patient's quality of life in the post-treatment period, it is important that health care providers encourage patients to explore their FP options before initiation of gonadotoxic treatment.^3,12,13^

The most notable provider-level deterrents described among Ontario physicians include not being sure where to refer women to as well as concerns regarding possible chemotherapy delay.^22^ Recent studies have demonstrated that postponing the commencement of chemotherapy in newly diagnosed breast cancer patients is often not necessary in the context of prompt FP referral and novel quick-start ovarian stimulation protocols.^15,39,40^ Furthermore, pursuit of FP does not appear to negatively influence prognosis.^40,41^ These encouraging findings need to be effectively conveyed to the health care providers involved in caring for AYA with cancer.

Our findings are consistent with prior studies that found age and parity to be significant determinants of FP referrals.^22,34,42,43^ The lower referral rates in older women with a prior live birth may be reflective of self-determined completion of childbearing or ambivalence about future childbearing. In the subset of older patients desiring future fertility, however, FP consult is imperative as they are more susceptible to gonadotoxicity. Advanced cancer stage has been consistently found to negatively impact referral rates.^8,44^ The majority of premenopausal cancer patients, however, have fertility concerns irrespective of age or prognosis.^18^

Regional trends in FP referrals were pronounced. Patients treated in south-east and south-west LHINs had significantly higher odds of having a fertility assessment before chemotherapy. The reduced odds of referral in patients managed at north LHIN sites did not remain statistically significant after multivariable analysis; however, this is likely attributed to the low overall number of consults rather a true lack of association. It has previously been described that the provincial distribution of new incident cancers roughly match the distribution of fertility centers.^45^ Certainly, the distance to a referral center may be a deterrent in more remote regions. Paradoxically, the south central region has the highest density of fertility clinics but has a lower proportion of FP referrals compared with the eastern and western LHINs. These regional disparities need to be more closely examined to further understand the systemic barriers to FP referrals in more centralized locations.

The medical specialty with the highest number of referrals was general surgery, followed by medical oncology and family medicine. Certainly, this distribution of FP referrals can be related to surgeons often being the primary point of care for breast cancer patients before the initiation of chemotherapy. Early involvement of the cancer care team may encourage prompt FP referrals from other subspecialists. The current literature on specialist referral patterns is limited to conflicting, small studies that are not amenable to comparison due to variations in care based on location practice and cancer type. Prior results based on survey data suggest that young age, female sex, and working in a multidisciplinary environment are physician characteristics positively associated with FP referrals.^6,46^

A limitation of this study is that gynecology consults in newly diagnosed cancer patients with a billing code for infertility were employed as a surrogate for FP referrals. Temporality increases the reliability of the measure as only consults occurring between cancer diagnosis and chemotherapy initiation were considered. Women with an infertility diagnosis before cancer were also excluded. Additionally, infertility was the most commonly billed diagnosis for pre-chemotherapy gynecologic consults. It is therefore unlikely that a large percentage of FP assessments were billed under another diagnosis. Another limitation is that the available data did not permit analysis of individual-level socioeconomic measures. As such, neighborhood income and marginalization index were employed as surrogates.

This study represents the first population-level assessment of FP referral rates and patterns in Canada. Despite recommendations that all pre-menopausal cancer patients be counseled on FP, referral rates remain low and continue to be influenced by patient demographic characteristics and cancer prognosis. Notably, determinants of FP referral need to be studied at a national level as population characteristics, health care coverage, and fertility clinic accessibility vary greatly across the country. Nonetheless, these findings highlight the need for further research aimed at overcoming these barriers to FP referral to adequately address the fertility concerns of newly diagnosed breast cancer patients in the province. Furthermore, future efforts in knowledge translation can facilitate health provider counseling on FP.
